# Impact of an educational intervention combining clinical obesity preceptorship with electronic networking tools on primary care professionals: a prospective study

**DOI:** 10.1186/s12909-020-02248-5

**Published:** 2020-10-14

**Authors:** Jean-Patrice Baillargeon, Denise St-Cyr-Tribble, Marianne Xhignesse, Christine Brown, André C. Carpentier, Martin Fortin, Andrew Grant, Judith Simoneau-Roy, Marie-France Langlois

**Affiliations:** 1 Division of Endocrinology, Department of Medicine, Université de Sherbrooke, Sherbrooke, Québec J1H 5N4 Canada; 2 Nursing school, Université de Sherbrooke, Sherbrooke, Québec J1H 5N4 Canada; 3 Department of Family Medicine, Université de Sherbrooke, Sherbrooke, Québec J1H 5N4 Canada; 4 Family Medicine Group, Centre Intégré Universitaire de Santé et de services sociaux du Saguenay-Lac St-Jean, Chicoutimi, Québec G7H 5H6 Canada; 5 Department of Biochemistry, Collaborative Research for Effective Diagnosis research unit, Université de Sherbrooke, Sherbrooke, Québec J1H 5N4 Canada; 6 Department of Pediatrics, Université de Sherbrooke, Sherbrooke, Québec J1H 5N4 Canada

**Keywords:** Continuing professional development, Team learning, Case-based learning, Online education, Preceptorship, Primary care, Obesity: lifestyle

## Abstract

**Background:**

Primary care providers’ (PCPs) attitude toward obesity is often negative, and their confidence level for helping patients manage their weight is low. Continuing professional development (CPD) on the subject of obesity is often based on a single activity using a traditional passive approach such as lectures known to have little effect on performance or patient outcomes. The aim of this study was to evaluate the impact of an educational intervention for obesity management on PCPs’ attitude, self-efficacy, practice changes and patient-related outcomes.

**Methods:**

Prospective interventional study with 12 months follow-up. A two-day clinical obesity preceptorship was offered where participants were actively involved in competence building using real-life situations, in addition to electronic networking tools, including a discussion forum and interactive monthly webinars. Thirty-five participants (12 nurses and 23 physicians) from seven Family medicine groups were enrolled. Questionnaires were used to evaluate the impact on primary care nurses’ and physicians’ attitudes and self-efficacy for obesity management. Practice changes and patient outcomes were evaluated using clinical vignettes, de-identified electronic patient records and qualitative analyses from group interviews.

**Results:**

Physicians’ general attitude towards patients with obesity was improved (61 ± 22 mm vs 85 ± 17 mm, *p* <  0.001). Self-efficacy for obesity management and lifestyle counselling were also improved immediately and 1 year after the intervention (all Ps <  0.05). De-identified patient records and clinical vignettes both showed improvement in recording of weight, waist circumference and evaluation of readiness to change lifestyle (all Ps <  0.05) that was confirmed by group interviews. Also, 15% of patients who were prospectively registered for weight management had lost more than 5% of their initial weight at the time of their last visit (*P* <  0.0001, median follow-up of 152 days).

**Conclusion:**

A multimodal educational intervention for obesity management can improve PCPs’attitude and self-efficacy for obesity management and lifestyle counselling. This translates into beneficial practice changes and patient-related outcomes.

**Trial registration:**

clinicaltrials.gov Identifier: NCT01385397. Retrospectively registered, 28 June 2011.

## Background

Obesity is a major public health problem recognized as an epidemic by the World Health Organisation [1]. It is associated with multiple co-morbidities, including type 2 diabetes, hypertension, cardiovascular disease and cancers [1]. According to data from the Canadian Health Measures Survey (2014–2015), 64.1% of the Canadian population now has overweight or obesity. This percentage has increased dramatically over the last 20 years [2].

Considering the growing prevalence of obesity in Canada, the majority of patients followed by primary care providers (PCPs) will have overweight or obesity [3, 4]. Studies have shown that many PCPs share negative stereotypes about people with obesity and often feel that they are unable to help their patients lose weight, their confidence level in obesity treatment being low [5–11]. As a result, obesity management in primary care is seldom undertaken compared to other chronic conditions like hypertension and diabetes [12–15]. Indeed, audits of medical records from PCPs show that obesity is underreported and recommendations for weight control interventions are infrequently followed [12, 16, 17].

Few studies have evaluated interventions aimed at changing health professionals’ attitudes, behaviours and organisation of care related to weight management [18]. Moore et al. reported that after a 4.5-h training program promoting obesity management, physicians that received the intervention were more likely to discuss and record weight than those in the control group [19]. Results from the Counterweight Program, a structured nurse-lead program with practice-based training and support, showed that 33% of patients followed in the program achieved a ≥ 5% weight loss at 12 months [20]. It is well known that continuing professional development (CPD) strategies based on a single activity using a traditional passive approach such as lectures are not associated with improvement in physicians’ performance or patient outcomes [21–24]. However, multiple interactive interventions in small groups over longer periods of time can translate into practice improvement [23, 25–27]. Indeed, it has been proposed that “*future CPD systems should be grounded in the workplace, integrated into the health care system, oriented to patient outcomes, guided by multiple sources of performance and outcome data (using the principles and strategies of quality improvement) and be team based under the collective responsibility of physicians, CPD providers, regulators and the health system”* [28].

As a means of engaging PCPs in obesity management, we developed obesity preceptorships in collaboration with the Continuing Medical Education (CME) office of the *Faculté de médecine et des sciences de la santé de l’Université de Sherbrooke.* Reviewing literature on effective CPD approaches, we chose to use preceptorships. This approach has been widely used in nursing [29–32] and it combines characteristics known to maximise practice change (multiple, small group interactive sessions in a health care environment) and improve health outcomes by enhancing provider self-confidence and competency [33–36]. The aim of this pilot study was to evaluate the impact of an educational intervention combining clinical obesity preceptorship with electronic networking tools. We particularly looked at PCPs’ attitudes, self-perceived general confidence for obesity management and self-efficacy for lifestyle counselling as well as related changes in clinical practice indicators (performance) and results (patient health), the latter two corresponding to levels 5 and 6 of Moore’s expanded CME framework [37]. Moore defines level 5 of his model as “*the degree to which participants do what the CME activity intended them to be able to do in their practices”* and level 6 as “*the degree to which the health status of patients improves due to changes in the practice behavior of participants”* [37]*.*

## Methods

### Setting

Family medicine groups (FMGs) have been implemented in Quebec since 2002 and are composed of groups of physicians who work closely with nurses in an environment that promotes an interdisciplinary approach to family medicine for registered patients [38]. In 2006, we approached FMGs within the territory of the *Integrated University Health Network* of the *Centre hospitalier universitaire de Sherbrooke* (CHUS) and the Faculty of Medicine and Health Sciences of the *Université de Sherbrooke*. This includes the Sherbrooke, Montérégie and Saguenay-Lac St-Jean regions in Quebec, Canada; Montérégie and Saguenay-Lac St-Jean are about 100 km and 500 km respectively from the CHUS in Sherbrooke.

### Study design and recruitment

The complete research protocol of this prospective interventional study was previously published [39]. Due to the lack of preliminary data for determination of sample size, we used a convenience sample of eight FMGs. From the 32 FMGs in the target region, eight out of 12 that were randomly contacted accepted to participate. There were no major differences between the practices that participated and those who declined. An FMG was eligible to participate if at least one primary care physician and one nurse were interested. All interested FMG team members were recruited with a maximum of six participants per FMG (48 participants being the capacity limit of our preceptorship program described below). One of these FMGs was targeted to become a key field informant and participated in the development and preliminary testing of the intervention components; it was not included in the analyses leaving seven FMGs for analysis.

The study was approved by the Human Research Ethics Committee of the CHUS and of the *Université de Sherbrooke* and all participants gave written informed consent before taking part in the study.

### Intervention

To design the intervention, we modified a pre-existing obesity preceptorship based on participants’ evaluations and needs assessment in accordance with clinical care norms. In addition, we developed web-based tools (for patients and professionals), including a discussion forum and interactive webinars. The final model consisted of a two-day preceptorship combining interactive sessions with content experts, case discussions and observation of real patient encounters with an interdisciplinary obesity management team from a tertiary care center (Table 1). The agenda was constructed such that theoretical knowledge could be reviewed before being exemplified through clinical practice (seeing real patients). This approach has the potential to effect change in professional practice, and, on occasion, health care outcomes [21–23, 25, 26]. The first day of the preceptorship was mandatory for all participants. It started with interactive sessions on initial patient evaluation, medical and surgical treatment options, behavioral approach to lifestyle modification and practical tips for diet and physical activity counseling; the rest of the day was dedicated to observation of real patient encounters as well as roundtable discussions using clinical vignettes. The second day, which occurred approximately 1 month later, was mandatory for nurses, but physicians could also attend. This day was designed to further address lifestyle counseling which was, in our integrated model, a task mainly delegated to the nurse although some physicians can engage in lifestyle counseling with positive results. The fact that there was a one-month time period between the first and second day of the preceptorship allowed participants to integrate any newly acquired knowledge/skills/attitudes into their practice routine thereby leaving an opportunity for discussion about difficulties encountered in practice. Pediatric obesity, nutritional approach and physical activity interventions were the themes of the interactive sessions of the second day and there were discussions of clinical vignettes at the end of the day.

**Table 1:** Preceptorship agenda

**DAY 1:**			
**Time**	**Activities**	**Educational modalities**	**Speaker**
8: 00 AM	Welcome and introduction		
8: 30 AM	CHUS Obesity Clinic functioning and results	IP	Endocrinologist/Nurse
8: 45 AM	Behavioural approach to lifestyle modifications and barriers to lifestyle modification	IP	Psychologist
9: 30 AM	Initial evaluation and objectives of weight loss	ICP	Endocrinologist
10: 00 AM	Health Break		
10: 15 AM	Complements of the initial evaluation	IP	Endocrinologist
11: 15 AM	Practical tips for physical activity intervention	IP	Kinesiologist
11: 30 AM	Practical tips for nutritional intervention	IP and PE	Dietitian
12: 00 PM	Lunch (with nutritional comments)		
12: 45 PM	Pharmacotherapy of obesity	IP	Endocrinologist
1:30 PM	CHUS obesity clinic: meetings with real patients	O	Endocrinologist
3:00 PM	Health break		
3:15 PM	Integration with clinical vignettes	CD	All preceptors
4:30 PM	Virtual community and Web site presentation	IP	Research assistant
5:15 PM	Evaluation and closing		
**DAY 2:**			
**Time**	**Activities**	**Educational modalities**	**Speaker**
8:00 AM	Welcome and introduction		
8:30 AM	Kids and Teenagers obesity	IP	Pediatric Endocrinologist
9:15 AM	Nutritional approach 1	IP and PE	Dietitian
10:30 AM	Health break		
10:45 AM	Nutritional approach 2	IP and PE	Dietitian
12:00 PM	Lunch (with nutritional comments)		
1:00 PM	Physical activity 1	IP	Kinesiologist
2:00 PM	Physical activity 2	EA and IP	Kinesiologist
2:30 PM	Patients’ teaching material presentation Return on virtual community portal	IP	Research assistant
3:15 PM	Health break		
3:30 PM	Integration with clinical vignettes	CD	All preceptors + Endocrinologist
5:15 PM	Evaluation and closing		

*IP* Interactive presentation; *ICP* Interactive case presentation; *EA* Experiential activity; *PE* Practical exercises; *O* Observation of real patient encounter; *CD* Case discussion

Participants had access to a website where they could find literature and tools for obesity management in addition to a discussion forum where they could interact with the tertiary care interdisciplinary obesity team on a continuous basis. After the preceptorship, monthly web meetings allowed participants to network with each other and interact with the tertiary care team. These interactive webinars included sessions on nutrition, physical activity and behavioural approaches to lifestyle modification, in addition to discussions of difficult cases submitted by participants. The study took place from 2006 to 2007.

### Outcome measures

Self-administered questionnaires using 100 mm visual-analog scales to measure attitude toward patients with obesity (1 mm = negative; 100 mm = positive), general confidence level for obesity management (1 mm = not confident; 100 mm = very confident) and perceived self-efficacy for physical activity and nutritional counseling (1 mm = not at all; 100 mm = totally) were completed. Two clinical vignettes featuring a patient with obesity (one consulting for an annual visit, the other specifically for obesity) were developed and used as proxies for measuring behaviour change in participants’ practice (Likert scale range from never (1) to always (5)). All participants completed the questionnaires before participating in the initial preceptorship, 1 month after the mandatory day of preceptorship (to measure immediate impact) and after 1 year (long-term impact).

A qualitative in-depth analysis of participants’ changes in their practice was performed using semi-structured group interviews in six FMGs 1 year after the preceptorship. The interview guide was based on the literature and drawn up by the research team [40]. The questions encouraged health care professionals to clarify their thoughts with regard to their experience with patients with obesity. Each group interview was digitally recorded, transcribed and coded to allow thematic analysis [41].

We also asked FMGs to complete de-identified electronic patient records for five consecutive unselected patients per participating physician, before their participation in the preceptorship and five other patients 1 year later. The data collected included anthropometric measures, vital signs, medication and major components of obesity management, In order to evaluate clinical outcomes of their weight management intervention, FMGs also completed a prospective electronic record for ten consecutive patients with obesity who were followed for weight management during the year following preceptorship.

### Data analysis

Normal distribution of continuous variables (including visual analog scale results) was ascertained using the Normal Quantile Plot. Continuous variables are reported as means ± SD and ordinal variables as medians with inter-quartile ranges (IQR). Baseline data were compared with data at 1 month and 1 year after the first day of preceptorship, within and between groups (MDs and nurses), using mixed model repeated measures ANOVA tests and *post-hoc* Tukey-Kramer tests. Changes in practice between baseline and 1 year after preceptorship were analyzed with Wilcoxon tests for the clinical vignettes and Fisher exact tests for the electronic patient records. Associations between perceptions and changes in practice were analyzed with Pearson’s correlation for continuous data or the Mann-Whitney test for dichotomous data. Data were analyzed using SPSS 17.0 and SAS for general linear mixed models to analyze repeated measures and longitudinal data.

All group interviews were independently coded by two researchers. Data were indexed, and the initial codes were grouped by properties or characteristics. Groupings were established among the categories when the investigators and collaborators judged the list of codes in the coding grids for the triads sufficiently exhaustive and meaningful [42].

## Results

We recruited 35 participants (12 nurses and 23 physicians). All participants attended the first day of the preceptorship while 77% (15/23 physicians and all 12 nurses) attended the second day. The following measures were used to evaluate the impact of our intervention:

### Attitude

**Fig. 1:**
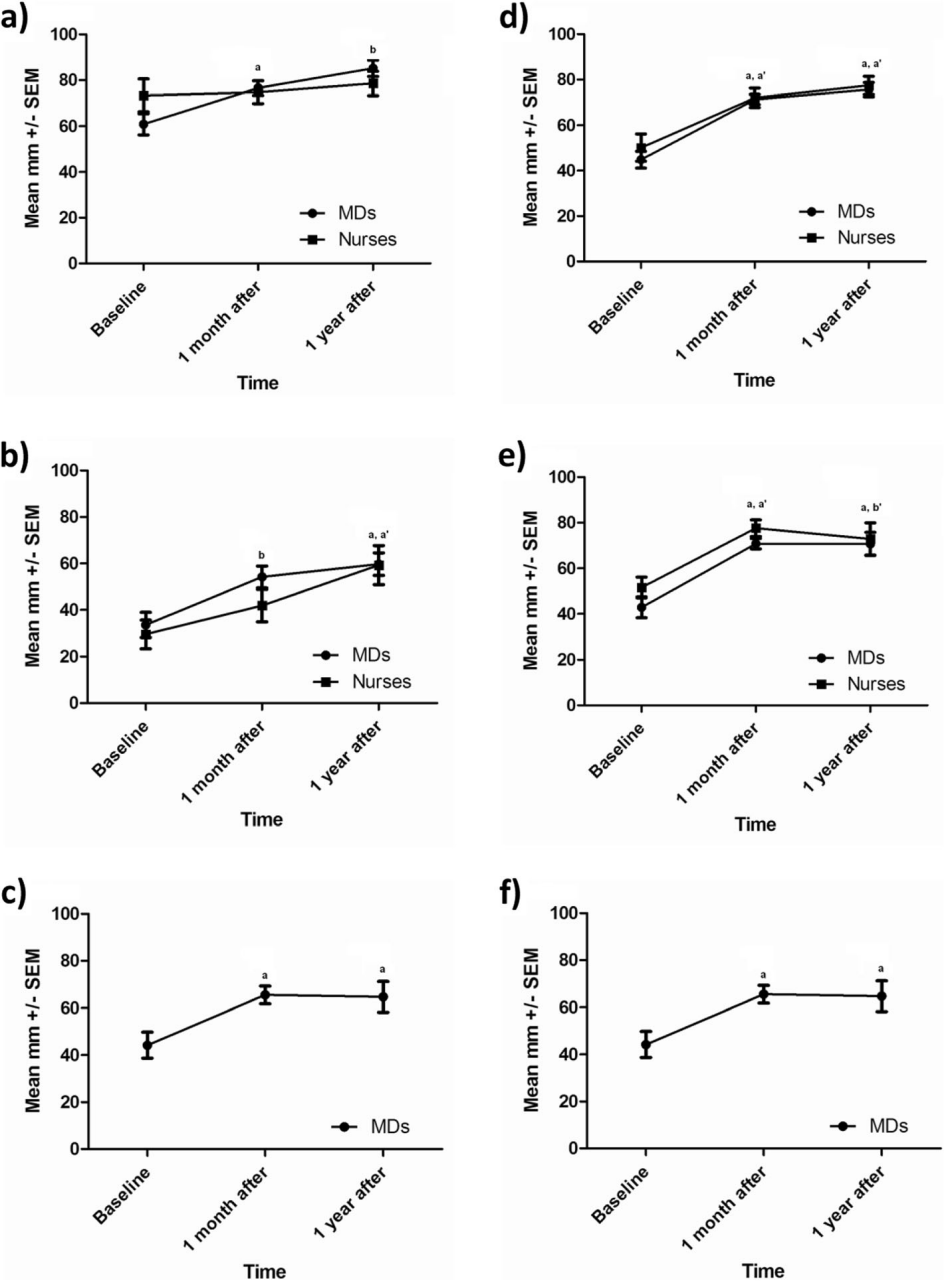
Doctors and nurses’ perceptions at baseline, 1 month and 12 months after the preceptorship. **a**. Attitude towards a patient with obesity. **b**. Confidence level to manage obesity. **c**. Confidence level to help adolescents or children manage their weight. **d**. Self-efficacy to give nutritional advice. **e**. Self-efficacy to give advice on physical activity. **f**. Self-efficacy to prescribe anti-obesity drugs*. a: *p* < 0.05 for MDs vs baseline; a’: *p* < 0.05 for nurses vs baseline; b: *p* < 0.001 for MDs vs baseline; b’:*p* < 0.001 for nurses vs baseline. * Only physicians’ data are presented because nurses do not prescribe medications.

Physicians’general attitude toward a patient with obesity was 61 ± 22 mm at baseline. This improved significantly after the preceptorship (77 ± 15 mm) and again at 1 year (85 ± 17 mm). Nurses’ attitude however did not change (Fig. 1a). There was no significant difference between physicians’ and nurses’ attitude toward a patient with obesity at baseline.

### General confidence level for the management of obesity

For all participants, confidence in the ability to manage obesity was low at baseline (48 ± 21 mm) but greatly and significantly increased after the preceptorship (72 ± 13 mm). This increased confidence level was maintained at 12 months in all participants (Fig. 1b). Confidence level to help adolescents or children manage their weight increased slightly after the preceptorship (from 34 ± 26 mm to 54 ± 22 mm for MDs and from 30 ± 21 mm to 42 ± 24 mm for nurses) but the change was significant only for physicians. This improvement tended to continue from one to 12 months after the initial training, such that both groups were significantly higher at 12 months (60 ± 23 mm for MDs and 60 ± 28 mm for nurses) compared to baseline (Fig. 1c).

### Self-efficacy for lifestyle counseling

Both groups increased significantly their self-efficacy to counsel patients regarding nutrition and physical activity at 1 month (from 48 ± 22 mm to 74 ± 12 mm for nutrition and from 54 ± 23 mm to 72 ± 16 mm for physical activity). This increase was maintained at 12 months (72 ± 24 mm for nutrition and 77 ± 19 mm for physical activity) (Fig. 1d-e). In addition, self-efficacy to prescribe anti-obesity medication increased significantly in MD participants after the preceptorship (from 44 ± 27 mm to 66 ± 17 mm) and was maintained at 1 year (Fig. 1f).

Finally, positive correlations were found between general confidence level to manage obesity and: i) improved attitude towards individuals with obesity (*p* = 0.001), ii) self-efficacy to give advice on physical activity (*p* = 0.022) and iii) confidence level to help children or adolescents manage their weight (*p* = 0.003).

### Self-reported changes in clinical practice

Table 2 presents changes in participants’ reported practice as measured by their responses to the clinical vignettes. Compared to baseline, participants reported an increased likelihood of offering systematic follow-up for obesity management to patients attending an annual preventive examination at 1 year. In the case of patients consulting specifically for a weight problem, significant changes in practice were numerous: the likelihood of evaluating readiness to change based on the Transtheoretical Model of Behavior Change [43, 44], offering systematic follow-up for obesity, using a food diary as well as suggesting the use of a pedometer significantly increased. We also found a positive correlation between self-efficacy to give nutritional advice and the reported use of a food diary (*p* = 0.026 for regular follow-up and *p* = 0.002 for obesity management vignette). Many practice changes related to obesity management were also documented in qualitative analysis of group interviews (Table 3).

**Table 2:** Changes in practice measured with clinical vignettes

	Patient with obesity consulting for annual exam		Patient with obesity consulting specifically for obesity	
	Median (IQR) Baseline	Median (IQR) 12 months	Median (IQR) Baseline	Median (IQR) 12 months
**Evaluation of readiness to change lifestyle**	3.00 (1.00)	4.00 (1.00)	4.00 (1.00)	5.00 (1.00)*
**Offer of weight management follow-up**	2.50 (1.00)	4.00 (1.00)*	4.25 (1.25)	5.00 (0.50)*
**Offer of group seminars**	2.00 (2.00)	1.00 (1.00)	3.00 (3.00)	1.00 (2.00)
**Use of food diary**	1.00 (1.00)	2.00 (2.00)	2.00 (2.00)	4.00 (1.75)*
**Referral to a dietician**	3.00 (1.00)	3.00 (1.75)	5.00 (1.00)	5.00 (1.00)
**Use of pedometer**	2.00 (2.00)	3.00 (1.00)	3.00 (3.00)	4.00 (1.00)*
**Offer/discuss antiobesity drugs**	1.00 (1.00)	1.00 (1.13)	3.00 (2.00)	2.00 (2.00)

*IQR* Interquartile range

*: *p* < 0.05 between baseline and 12 months; Wilcoxon signed ranks test

**Table 3:** Reported practice changes during group interviews

Reasons given	Indicators	Quotes
Development of an obesity management procedure	Recognition of obesity as a medical problem	*“I was doing it less often before, I write obesity or overweight in my* (medical) *problem list.”*
	Measures of anthropometric data and history of weight	*“I do it* (waist circumference) *now in almost all patients who come to my office”* *“Like all our follow-up, we will weight them more, calculate the body mass index...”*
	Weight management is part of the usual intervention	*“It’s a subject that I will approach more easily than before.”* “*It also brought a focus on the treatment of obesity in the clinic which was not present before”*
	Realistic weight loss goals	“(I tell my patients) *to aim for 5 to 10% of weight loss…”* “*You will not be back to your healthy weight but if you lose 10% already it is meaningful for your health.”*
	Frequent appointments	*“For most* (patients with obesity), *I see them every four weeks.”*
	Weight maintenance is a success in some patients	*“This is an argument I did not have: you're not gaining* (weight) *but at least you are maintaining it.”* *... they do more exercise than before and have better eating habit, they can be winners. They will certainly maintain their weight.*
	Increased physical activity counselling	*“Walking, I talk about it all the time”*
	Use of food diary	“*Using the food diary, it was something that was frightening me ….. but I tried it with some patients and I saw that we could carry on with that and it was beneficial.”* *“I used the food diary a lot.”*

### Impact on clinical assessment and patient outcomes

An electronic chart review of randomly selected patients was performed by the FMG professionals themselves at baseline and at 1 year, and several significant changes between baseline and at 1 year were documented by participants (Table 4): PCPs measured the weight of their patients and their waist circumference and evaluated readiness for change in their patients more often. No change was observed in other actions such as blood pressure measurement or listing of medications that were used as controls for a reporting bias.

**Table 4:** Practice changes estimated from electronic chart review of patients

Information	% of charts that reported the information before preceptorship (***n*** = 55)	% of charts that reported the information one year after the preceptorship (***n*** = 53)	***p***-value*
**Evaluation of readiness to change lifestyle**	12.7% (7)	32.1% (17)	< 0,05
**Weight**	63.6% (35)	83.0% (44)	< 0,05
**Waist circumference**	7.3% (4)	35.8% (19)	< 0,0001
**Blood pressure**	74.5% (41)	84.9% (45)	NS
**Medication listing**	72.7% (40)	52.8% (28)	NS

*Fisher’s exact test

**Fig. 2:**
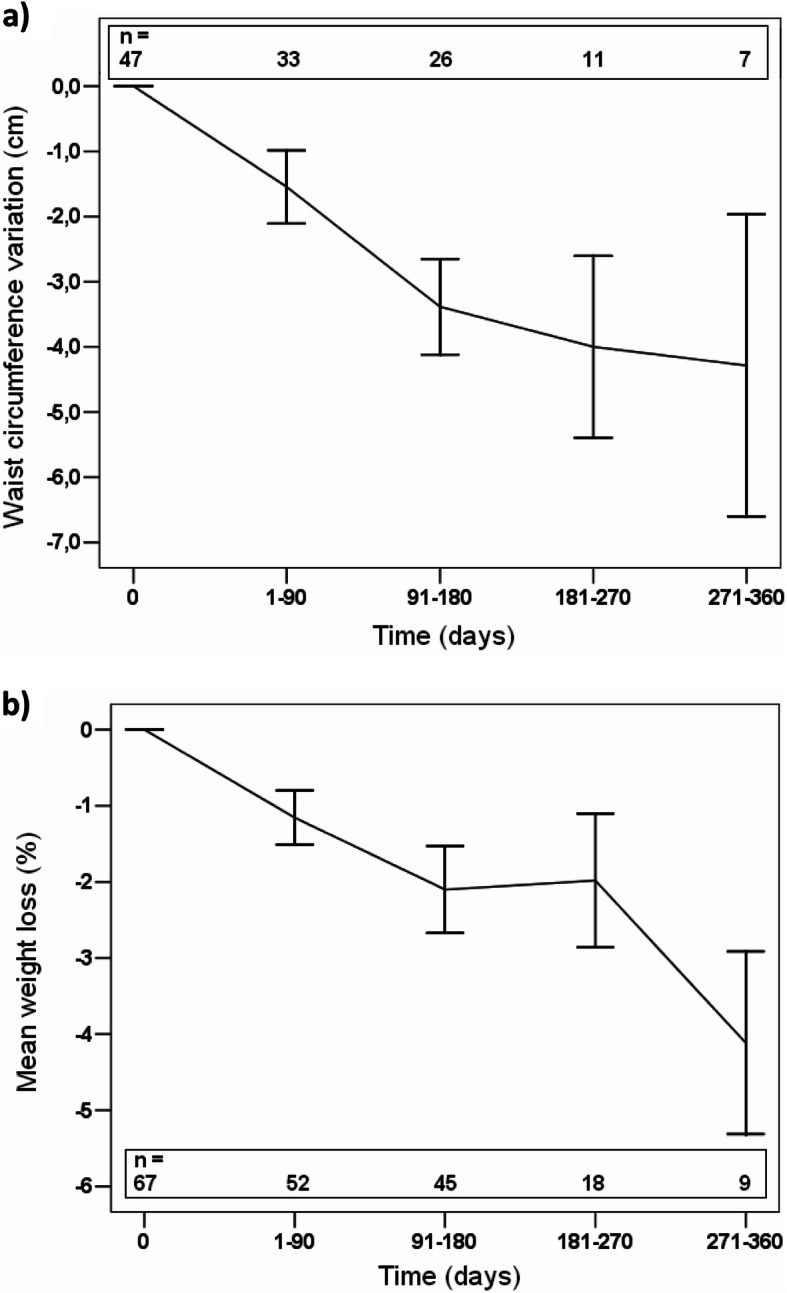
Variation in waist circumference and percentage of weight loss as recorded in the prospective electronic patient record (Mean ± SEM)

Moreover, in the patients that our participants followed prospectively for weight management, we observed significant decreases in waist circumference (− 4.3 ± 6.1 cm, *P* <  0.0001) and weight (− 4.5 ± 4.3 kg, *P* <  0.0001). Figure 2 shows that 15.2% of these patients lost 5% or more of their initial weight during their follow-up (median follow-up of 152 days) and that they significantly reduced their waist circumference.

## Discussion

This study confirms the low confidence level of PCPs in dealing with patients with obesity as well as existing gaps in the management of obesity in primary care. It also demonstrates that training PCPs from FMGs and providing them with the support of electronic networking tools improves their attitude toward patients with obesity, their confidence level for obesity management and their self-efficacy to perform lifestyle counselling. Furthermore, it significantly improves some clinical practice indicators as measured by clinical vignettes and audit of electronic patient records.

In a national survey among Canadians, 30% of individuals with overweight or obesity reported that their physicians had advised them to lose weight [45]. Low confidence level for obesity management may be an important barrier to implementing interventions in clinical practice [7, 10, 46, 47]. Our findings are similar to those of a survey of 620 primary care physicians in which 92% of respondents considered obesity as a chronic disease but only 49% felt confident to treat it [7]. This survey also reported a high prevalence of negative perception of patients with obesity as more than 50% of physicians viewing these patients with negative attributes [7]. Results of our study suggest that a multimodal educational intervention for obesity management can succeed in changing the negative bias towards individuals with obesity and the low confidence level of physicians to treat them.

According to the Canadian Clinical Practice Guidelines [48], diagnosing obesity is the first step in evaluating a patient. Baseline data from our electronic chart review of patients are similar to those of a study auditing medical records from PCPs with an interest in managing obesity, which showed that body mass index (BMI) was recorded for only 64.2% of patients [49]. In a recent survey, only 14% of patients reported having their waist circumference measured in the last 12 months compared to our results which showed that waist circumference was reported in 35% of medical charts [45]. Our data from electronic patient records also indicate that PCPs more frequently reported the weight and waist circumference of their patients after the educational intervention. Moreover, we demonstrated improvement in other indicators of effective obesity management (evaluation of readiness to change, use of self-monitoring tools) and an association between these and the self-efficacy of health professionals. These changes were confirmed by results from our semi-structured group interviews.

A review published in 2017 on trials evaluating the effect of educational interventions aimed at general practitioners versus standard care found that educating health professionals, using traditional CPD approaches, is usually associated with small reductions in patients’ weight (− 1.24 kg) [18]. Our study found a greater impact on patients’ weight (− 4.5 kg), with 15% of patients losing at least 5% of their weight during the time of the study; this is a clinically significant weight loss goal known to be associated with reduction of co-morbidities [50–54]. Perhaps our results represent the use of more evidence-based educational strategies. Results from the “Counterweight Programme” also suggest that empowerment and education of health professionals and patients in primary care settings can have a successful impact on modification of their health behaviour and weight [55].

One major strength of this work is the assessment of the long term effects of our educational and networking interventions. One-year measurements provide insight as to whether participants were simply trying out new skills or whether they had also incorporated these as a regular feature of their clinical practice (a measure of deep learning). Our educational approach was based on active learning principles that can easily be transferred to other settings. A variety of tools were used to evaluate perceptions and clinical practice in order to triangulate data. However, a weakness of the study arises from the fact that most evaluations are self-reported and may be affected by desirability biases. It is also possible that the participants selected a biased patient cohort for electronic patient records. However, because participants were instructed to enter consecutive patients and that patients’ data was entered prospectively from the start of the intervention, the potential for selection bias was probably minimized. Furthermore, few predictors of patient success, allowing for selection of patients more likely to lose weight, were known at that time [56]. For the purpose of this study, participants had to enter the clinical data (blood pressure, weight, waist circumference, etc.) in another system thus duplicating the recording of clinical data. This could have contributed to some of the low level of blood pressure or weight reported at each visit which may be underreported. The absence of a control group and direct patient evaluations are also limitations of the study.

## Conclusion

In summary, obesity preceptorships conducted specifically in the context of a multimodal educational intervention for obesity management coupled with networking electronic tools changed perceptions of health professionals and improved reported management of obesity by PCPs in FMGs after 1 year. Further studies are required to evaluate the efficacy of such educational intervention for obesity management.

## Data Availability

The datasets used and/or analysed during the current study are available from the corresponding author on reasonable request.

## Abbreviations

BMI: Body mass index
CHUS: Centre hospitalier universitaire de Sherbrooke
CME: Continuing medical education
CPD: Continuing professional development
FMG: Family medicine group
IQR: Inter-quartile ranges
PCP: Primary care provider
SD: Standard deviation
